# A task is a task is a task: putting complex span, *n*-back, and other working memory indicators in psychometric context

**DOI:** 10.3389/fpsyg.2014.01475

**Published:** 2014-12-23

**Authors:** Florian Schmiedek, Martin Lövdén, Ulman Lindenberger

**Affiliations:** ^1^Center for Lifespan Psychology, Max Planck Institute for Human Development, Berlin, Germany; ^2^German Institute for International Educational Research, Frankfurt, Germany; ^3^Aging Research Center, Karolinska Institutet and Stockholm University, Stockholm, Sweden

**Keywords:** working memory, latent factors, psychometrics, complex span, *n*-back, memory updating

## Abstract

Based on a meta-analysis, Redick and Lindsey (2013) found that complex span and *n*-back tasks show an average correlation of *r* = 0.20, and concluded that “complex span and *n*-back tasks cannot be used interchangeably as working memory measures in research applications” (p. 1102). Here, we comment on this conclusion from a psychometric perspective. In addition to construct variance, performance on a test contains measurement error, task-specific variance, and paradigm-specific variance. Hence, low correlations among dissimilar indicators do not provide strong evidence for the existence, or absence, of a construct common to both indicators. One way to arrive at such evidence is to fit hierarchical latent factors that model task-specific, paradigm-specific, and construct variance. We report analyses for 101 younger and 103 older adults who worked on nine different working memory tasks. The data are consistent with a hierarchical model of working memory, according to which both complex span and *n*-back tasks are valid indicators of working memory. The working memory factor predicts 71% of the variance in a factor of reasoning among younger adults (83% for among older adults). When the working memory factor was restricted to any possible triplet of working memory tasks, the correlation between working memory and reasoning was inversely related to the average magnitude of the correlations among the indicators, indicating that more highly intercorrelated indicators may provide poorer coverage of the construct space. We stress the need to go beyond specific tasks and paradigms when studying higher-order cognitive constructs, such as working memory.

## INTRODUCTION

Cognitive psychology is interested in constructs such as working memory, selective attention, or memory retrieval. Theoretically, constructs are defined by a set of mechanisms, or cognitive processes. Empirically, researchers get at constructs by observing individuals’ behavior on specific tasks or paradigms (e.g., sets of similar tasks). When doing so, researchers commonly agree that no task or paradigm ever, as valid as it might be, is process-pure; rather, in addition to the processes of interest, a host of task- and paradigm-specific processes contribute to performance.

Using the same task or paradigm within or across experiments holds unwanted sources of variance constant, and thereby helps in delineating the effects of experimental manipulations. Nevertheless, the generalizability of results to the construct level increases considerably if researchers use different tasks and paradigms. One particularly powerful method to find out whether research is indeed making progress toward identifying and characterizing a hypothesized construct is to check whether individual differences in performance on different tasks assumed to index the same construct correlate with each other. If they do not, this should be taken as a warning signal that researchers might be using tasks that tap different theoretical constructs to begin with or that they are using tasks dominated by task-specific variance, paradigm-specific variance, measurement error, or a combination of all three. Hence, when correlations among tasks assumed to measure the same construct are low, this phenomenon deserves further scrutiny.

In research on working memory, a variety of paradigms is currently in use. In addition to the well-established complex span tasks, which are basically dual tasks that require memorizing a list of items (e.g., words) while making simple decisions (e.g., verifying equations), the *n*-back paradigm (Kirchner, 1958; Cohen et al., 1997) has been used extensively, particular in the fields of neuroscience, clinical, and aging research. For the overarching aim of better understanding working memory, this parallel existence of two often used kinds of tasks makes it important to confirm that both are measuring the same underlying construct, that is, have good construct validity. For the complex span task *operation span* (Turner and Engle, 1989) and a letter *n*-back task, Kane et al. (2007) reported weak correlations in the range of 0.20, and questioned the construct validity of the *n*-back task. Since then, several studies have reported correlations of complex span and *n*-back tasks and, recently, Redick and Lindsey (2013) took the effort to conduct a meta-analysis to integrate the wide range of correlations that have been observed thus far (e.g., from –0.07 to +0.50). The meta-analytically estimated mean correlation was 0.20. Based on this estimate, the authors concluded that complex span and *n*-back tasks must not be used interchangeably as indicators of a common working memory construct.

Low correlations between tasks can result from a number of reasons. First, the tasks can really measure different constructs. Second, individual differences in tasks might be dominated by task-specific sources of variance. These sources of variance might be further differentiated into sources that are specific to paradigms (e.g., the possibility to use of familiarity information in *n*-back tasks; Schmiedek et al., 2009b) and sources that are specific to contents (e.g., the requirement to count quickly in a counting span task). Third, measurement error and restrictions of range (e.g., floor or ceiling effects) might lower correlations. Before interpreting low correlations between tasks as indicating that they measure different constructs, these sources of variance must be separated. Fortunately, these different possibilities (with the exception of restrictions of range) can be comprehensively disentangled if tasks are (1) put into a psychometric context of tasks that represent different paradigms and task contents; and (2) analyzed with data-analytic approaches, such as confirmatory factor analysis (CFA), which allow for separating shared and unique sources of variance at different levels of a hierarchy from each other as well as from measurement error.

With the aim of identifying the shared variance of complex span tasks and tasks that were broadly classified as updating tasks of working memory, Schmiedek et al. (2009a) showed that a latent factor of complex span tasks (reading span, counting span, and rotation span) correlated 0.97 with the factor of updating tasks (numerical memory updating, alpha span, spatial *n*-back). This result shows that, once measurement error and task-specific sources of variance were accounted for, the shared variance of different complex span tasks was identical to the shared variance of different updating tasks. Because paradigms and contents were confounded across the three updating tasks (i.e., each paradigm was operationalized with only one content), however, it was not possible to draw further conclusions about whether the task-specific variance was due to the different paradigms or the different contents of the tasks.

Just as complex span can be operationalized in numerous ways (i.e., by combining different to-be-memorized contents with all kinds of secondary decision tasks), it is possible to operationalize the different updating paradigms used by Schmiedek et al. (2009a) with different contents. For the present investigation, we propose the following classification of paradigms^1^. First, the *memory updating* paradigm (Salthouse et al., 1991) comprises tasks in which several elements (e.g., digits or spatial positions) have to be stored and then simultaneously be updated according to a series of operations (e.g., arithmetic operations or spatial movements), before the end results have to be recalled. Second, *sorting span* tasks require the storage of a list of elements (e.g., letters or objects) and the simultaneous ordering of them according to some dimension (e.g., alphabetical order or size). Third, *n*-back tasks require permanently updating memory to store the last n elements (e.g., digits or spatial positions) of a sequence and make decisions as to whether the most recent element matches that one *n* steps back in the sequence. What is common to all three paradigms is that they all require simultaneous storage and processing, that is, working memory as commonly defined (e.g., Baddeley, 2007). What makes them different could be a number of things, including the applicability of different strategies (e.g., Shing et al., 2012), the different degree to which familiarity information might be used (Oberauer, 2005), the different degrees to which shifting the focus of attention is required (Oberauer, 2003), and the involvement of retrieval processes from long-term memory (Unsworth and Engle, 2007).

Within each paradigm, the number of tasks that one could create by varying task content is potentially large and further introduces sources of variance, like differential expertise with necessary basic skills (e.g., mental calculus), differential knowledge (e.g., about placement of objects along a dimension like size), and the applicability of certain strategies (e.g., visualization). Even if each task was measured with perfect reliability, the observed correlations between two single tasks therefore need not be high—and still, they both might be valid indicators of working memory (i.e., the task vectors may point to the same centroid in construct space; see Figure 1 in Little et al., 1999).

**FIGURE 1:**
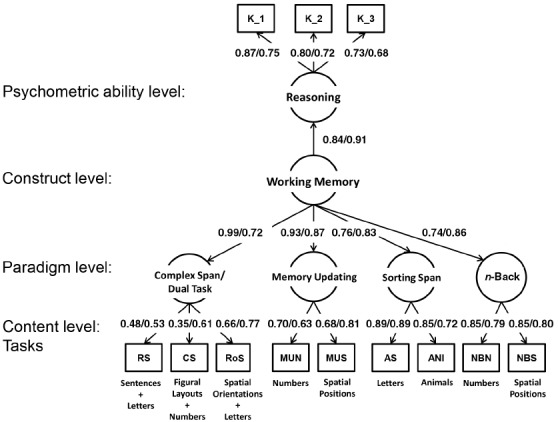
**Structural equation model with latent factors (circles) for different paradigms measuring the underlying latent construct of working memory.** Working memory predicts a latent factor of reasoning. All factor loadings and latent regression paths are standardized. Left values are for younger adults, right values are for older adults. RS, reading span; CS, counting span; RoS, rotation span; NBN, *n*-back numerical; NBS, *n*-back spatial; MUN, memory updating numerical; MUS, memory updating spatial; AS, alpha span; ANI, animal span; K_1, reasoning, Parcel 1 (BIS test); K_2, reasoning, Parcel 2 (BIS test); K_3, reasoning, Parcel 3 (BIS test).

The aim of the present investigation was to replicate the findings of Schmiedek et al. (2009a) with additional samples, and to disentangle the influence of using different paradigms, and different contents within paradigms, on the size of correlations between tasks. In addition to complex span (i.e., reading span, counting span, rotation span) and *n*-back tasks (i.e., spatial and letter 3-back), we also considered sorting span (i.e., alpha span and animal span) and memory updating tasks (i.e., numerical and spatial memory updating) to arrive at a comprehensive picture of different paradigms. The model we propose for this comprehensive psychometric perspective is a hierarchical structure with a general working memory factor on top (i.e., the construct level), operationalized with different paradigms (i.e., the paradigm level), which in turn are measured with tasks of different content (i.e., the content level; see Figure 1). Because of the prominence of the *n*-back task in cognitive aging research and because, generally, different paradigms might not work equally well for different age groups, we included samples of older and younger adults in our investigation. Finally, we also investigated the relations of the working memory factor to a latent factor of reasoning to validate the working memory factor with a well-established construct in the space of intellectual abilities (e.g., Carroll, 1993). The data sets were taken from the pretest of the COGITO Study (for details, see Schmiedek et al., 2010b).

## MATERIALS AND METHODS

### PARTICIPANTS

One-hundred and one younger (51.5% women, age 20–31 years) and 103 older adults (49.5% women, age 65–80 years) participated in the study. Older adults were screened for cognitive deficits with the MMSE at pretest and posttest of the COGITO study, resulting in scores of 25 or higher on at least one of the occasions for all older participants. Further details about sample characteristics and study dropout can be found in Schmiedek et al. (2010a,b).

### PROCEDURE

Before entering a longitudinal phase of 100 daily testing sessions, participants completed a pretest of 10 sessions that comprised 2–2.5 h of comprehensive cognitive test batteries and self-report questionnaires. The tasks in the present investigation were distributed over seven of these sessions. Participants worked on the tasks in lab rooms with up to six workstations. To individualize task difficulty for the longitudinal training phase, presentation rates for the 3-back spatial, Memory updating numerical, and Alpha span tasks were fixed for each participant to one out of three possible rates based on pretest performance (see Schmiedek et al., 2010b for details). Because only one presentation rate condition was used for the WM tasks that were not part of the training phase, only the corresponding condition was used for the practiced tasks in our analyses.

Participants were paid between 1450 and 1950 EUR, depending on the number of completed sessions and their pace of completing the longitudinal phase of the study.

#### Complex span tasks

Three complex span tasks were included in one of the pretest sessions. Those were *reading span* (Daneman and Carpenter, 1980), *counting span* (Case et al., 1982), and *rotation span* (Shah and Miyake, 1996).

***Reading span.*** We used a version that differed from the original version in that participants did not have to memorize words but single letters (cf. Kane et al., 2004). Several sentences were presented successively. Below each sentence, a letter was displayed. Participants had to decide whether the sentences were semantically correct within up to 10 s, to memorize the letter, and, after a sequence of sentence–letter combinations, recall the letters in their order of presentation. Twelve blocks of trials, three for each load-level (of 2–5) were included.

***Counting span.*** Our version of counting span was similar to the one used by Kane et al. (2004). Several displays of blue circles (4–9), green circles (1–5), and blue squares (1–9) were presented. Participants had to count the blue circles and make decisions as to whether the number was odd or even within up to 10 s. The numbers of blue circles had to be memorized for later recall in the order of their presentation. The number of displays ranged from 2 to 6 per block of trials. A total of 15 blocks was completed, three per load-level.

***Rotation span.*** This task combines recall of a sequence of short and long arrows, radiating from the center of the display, with a letter-rotation task (Kane et al., 2004; Wilhelm and Oberauer, 2006). First, a regular or mirror-reversed letter (rotated by 0–315°) was displayed. The processing requirement was to decide whether letters were displayed regularly or mirror-reversed within up to 5 s. After each processing step (ranging from two to five per block), short or long arrows were shown, pointing in one of the eight directions. At the end of one sequence, participants had to recall the direction and length of the arrows in the order of their presentation and indicate them by clicking on a layout with the 16 possible positions of the arrow head. There were 12 blocks of trials to complete, three per load-level.

#### *N*-back tasks

Two versions of a 3-back task, one numerical and one spatial were included.

***3-Back spatial.*** A sequence of 39 black dots appeared at varying locations in a 4 by 4 grid. Participants were supposed to recognize whether each dot was in the same position as the dot three steps earlier in the sequence or not. Dots appeared at random locations with the constraints that (a) 12 items were targets, (b) dots did not appear in the same location in consecutive steps, (c) exactly three items each were 4-, 5-, or 6-back lures, that is, items that appeared in the same position as the items 4, 5, or 6 steps earlier. No lures of lags longer than 6 were included. The presentation time for the dots was 500 ms. ISI was 500, 1500, 2500, or 3500 ms, resulting in a fixed presentation rate of 1, 2, 3, or 4 s. For the present analyses, only the eight blocks with ISI of 2500 ms were used, across which accuracy was averaged.

***3-Back numerical.*** As in the spatial version of the 3-back, two-choice decisions on whether the current stimulus matches the stimulus shown three steps earlier in the sequence had to be made. Instead of spatial positions, the 39 stimuli were one-digit numbers (1–9). PT was 3000 ms with an ISI of 1000 ms, resulting in a fixed presentation rate of 4000 ms. Six blocks were conducted in total. Average accuracy was used as the performance score.

#### Memory updating tasks

Two memory updating tasks, one numerical and one spatial, were included.

***Memory updating numerical.*** Four single digits (ranging from 0 to 9) were presented simultaneously in four cells situated horizontally for 4000 ms. After an ISI of 500 ms, a sequence of eight updating operations was presented in a second row of four cells below the first one. These updating operations were additions and subtractions within a range of –8 to +8. Those updating operations had to be applied to the digits memorized from the corresponding cells above and the updated results had to be memorized. Each updating operation was applied to a different cell from the one a step earlier in the sequence, so that no two updating operations had to be applied to one cell in a sequence. Presentation time was varied with 12 blocks each of 500, 1250, 2750, and 5750 ms. ISI was 250 ms, resulting in a fixed presentation rate or 750, 1500, 3000, or 6000 ms. At the end of each trial, the four end results had to be entered in the four cells in the upper row. All intermediate and end results ranged between 0 and 9. For the present analyses, only the 12 blocks with ISI of 2750 ms were used, across which accuracy was averaged.

***Memory updating spatial.*** In each block of this task, first a display of four 3 × 3 grids was shown for 4000 ms in each of which one black dot was present in one of the nine locations. Those four locations had to be memorized and updated according to shifting operations, which were indicated by arrows appearing below the corresponding field. Presentation time of the arrows was 2750 ms with an ISI of 250 ms, resulting in a fixed presentation rate of 3000 ms. After six updating operations, the four grids reappeared and the resulting end positions had to be clicked on. After 12 practice blocks with memory load 2, six test blocks with load 2, six test blocks with load 3, and 12 test blocks with load 4 were conducted and the average accuracy used for scoring.

#### Sorting span tasks

Two versions of sorting span were included, one using the alphabetical order of letters and one ordering animal names by the size of the animals.

***Alpha span.*** In our adapted version of the original Alpha span by Craik (1986), 10 upper-case consonants were presented sequentially together with a number below the letter. For each letter, participants had to decide as quickly as possible whether the number corresponded to the position of the current letter in the alphabetically ordered set of letters presented up to this step. For example, if after a first letter-digit combination of “K-1” (which necessarily always was correct), the combination “P-2” was shown, participants had to respond “Correct,” because P is the second letter in the correctly ordered sequence “K, P.” If “B-2” was presented next, participants should respond “Incorrect,” because B is the first (and not the second) letter in the accumulated ordered sequence “B, K, P,” and so forth. Five of the 10 items were targets. If the position numbers were incorrect (non-targets) they differed from the correct position by ±1. The presentation time for the letters was individually adjusted based on pre-test performance. Presentation time was varied with 12 blocks each of 750, 1500, 3000, and 6000 ms. ISI was 500 ms, resulting in a fixed presentation rate of 1250, 2000, 3500, or 6500 ms. For the present analyses, only the 12 blocks with ISI of 3000 ms were used, across which accuracy was averaged.

***Animal span.*** As in the alpha span task, a list of consecutively shown stimuli had to be ordered continuously. Instead of letters, six names of animals were shown one after the other, which had to be ordered by size and two-choice decisions on whether a given number corresponds to the current rank order of the present animal had to made. Presentation time was 3000 ms with an ISI of 1000 ms, resulting in a fixed presentation rate of 4000 ms. Eight blocks were conducted in total.

#### Reasoning tasks

From the reasoning scale of the BIS test (Jäger et al., 1997; for English descriptions, see Carroll, 1993; Wilhelm and Schulze, 2002; Süß and Beauducel, 2005) nine reasoning items (three for each content category—verbal, numerical, and figural) were used. The nine tasks were z-standardized and aggregated into three parcels that served as indicator variables for the latent reasoning factor. Each parcel consisted of one verbal, one numerical, and one figural task.

### DATA ANALYSIS

To apply the hierarchical factor model, a structural equation modeling approach using Mplus 7 with ML estimation was used. Multiple-group models were used to test for configural and metric measurement invariance (Vandenberg and Lance, 2000) across age groups.

## RESULTS

### PRELIMINARY ANALYSES

Descriptive statistics for all tasks are reported in Table 1. Internal consistencies for the working memory tasks were satisfactory to very high (Cronbach’s alpha: range 0.71–0.95; see Table 1). Correlations among tasks did vary considerably, from 0.09 to 0.75 in the younger sample and from 0.14 to 0.67 in the older sample (see Table 2). The highest correlations were observed for tasks belonging to the same paradigms, while the lowest correlations were found between reading span and tasks from the *n*-back (younger adults) or sorting span paradigms (older adults). These comparatively low correlations cannot simply be explained with the comparatively low reliability of reading span. Even assuming perfect reliability, the correlation of reading span and *n*-back numerical, for example, would only be 0.11 (correction for unreliability: *r* = 0.09/(0.71 × 0.92)^1/2^. Given the generally high internal consistencies, the difference in size of the correlations has to be primarily due to systematic task- and paradigm-specific sources of variance, which will be disentangled below, using structural equation modeling.

**Table 1:** Descriptive statistics and Cronbach's Alphas.

**Variable**	**No. of blocks**	***M* YA/OA**	**SD YA/OA**	**Skew YA/OA**	**Kurtosis YA/OA**	***α YA/OA***
RS	12	0.87/0.80	0.11/0.14	–1.22/–0.81	0.88/0.35	0.71/0.77
CS	15	0.85/0.74	0.16/0.12	–3.16/–0.49	14.02/–0.08	0.90/0.72
RoS	12	0.82/0.54	0.13/0.15	–1.52/–0.44	4.04/–0.43	0.77/0.75
NBN	6	0.89/0.75	0.09/0.10	–0.86/–0.17	0.09/–0.09	0.92/0.92
NBS	8	0.85/0.70	0.11/0.10	–0.91/–0.04	0.13/–0.46	0.95/0.95
MUN	12	0.79/0.58	0.17/0.21	–1.32/–0.36	2.17/–0.22	0.85/0.88
MUS	24	0.64/0.43	0.16/0.13	0.25/0.04	–0.41/–0.41	0.91/0.84
AS	12	0.73/0.60	0.09/0.08	–0.35/0.05	1.01/–0.58	0.81/0.81
ANI	8	0.84/0.57	0.12/0.13	–1.94/0.54	6.41/0.04	0.84/0.76

RS, reading span; CS, counting span; RoS, rotation span; NBN, n-back numerical; NBS, n-back spatial; MUN, memory updating numerical; MUS, memory updating spatial; AS, alpha span; ANI, animal span; α, internal consistencies (Cronbach's alpha); YA, younger adults; OA, older adults.

**Table 2:** Correlations among all tasks.

	**RS**	**CS**	**RoS**	**NBN**	**NBS**	**MUN**	**MUS**	**AS**	**ANI**	**K_1**	**K_2**	**K_3**
RS	–	0.67*	0.40*	0.33*	0.27*	0.34*	0.35*	0.14	0.22*	0.21*	0.24*	0.17
CS	0.40*	–	0.44*	0.38*	0.32*	0.47*	0.32*	0.19	0.13	0.23*	0.21*	0.16
RoS	0.31*	0.29*	–	0.41*	0.38*	0.36*	0.41*	0.38*	0.26*	0.33*	0.34*	0.25*
NBN	0.09	0.27*	0.43*	–	0.66*	0.42*	0.57*	0.49*	0.43*	0.48*	0.57*	0.37*
NBS	0.15	0.23*	0.49*	0.69*	–	0.35*	0.46*	0.52*	0.36*	0.50*	0.50*	0.39*
MUN	0.45*	0.34*	0.40*	0.47*	0.48*	–	0.50*	0.36*	0.33*	0.37*	0.37*	0.32*
MUS	0.32*	0.36*	0.54*	0.35*	0.41*	0.51*	–	0.45*	0.33*	0.39*	0.37*	0.43*
AS	0.26*	0.29*	0.40*	0.42*	0.37*	0.32*	0.45*	–	0.63*	0.53*	0.50*	0.52*
ANI	0.27*	0.18	0.39*	0.31*	0.31*	0.36*	0.41*	0.75*	–	0.47*	0.47*	0.44*
K_1	0.36*	0.27*	0.45*	0.29*	0.39*	0.42*	0.48*	0.59*	0.56*	–	0.58*	0.48*
K_2	0.39*	0.24*	0.38*	0.34*	0.32*	0.43*	0.52*	0.61*	0.59*	0.67*	–	0.46*
K_3	0.30*	0.26*	0.36*	0.17	0.28*	0.35*	0.44*	0.50*	0.52*	0.69*	0.56*	–

Younger adults below the diagonal (N = 101), older adults above the diagonal (N = 103); RS, reading span; CS, counting span; RoS, rotation span; NBN, n-back numerical; NBS, n-back spatial; MUN, memory updating numerical; MUS, memory updating spatial; AS, alpha span; ANI, animal span; α, internal consistencies (Cronbach's alpha); K_1, reasoning, Parcel 1 (BIS test); K_2, reasoning, Parcel 2 (BIS test); K_3, reasoning, Parcel 3 (BIS test).

*p < 0.05.

### LATENT-VARIABLE ANALYSES

A higher-order factor model for working memory as shown in Figure 1 was fit to both age groups simultaneously using multi-group structural equation modeling. Model fit of a model with configural measurement invariance across age groups was satisfactory [Model 1: χ^2^(44) = 63.9, CFI = 0.97, RMSEA = 0.07, SRMR = 0.05]. In this and all subsequent models, correlated residuals of reading span and counting span were allowed based on modification indices. Constraining factor loadings of tasks on paradigm factors to be equal across age groups did neither reduce model fit descriptively [Model 2: χ^2^(49) = 69.3, CFI = 0.97, RMSEA = 0.06, SRMR = 0.07], nor by statistical criteria [Δχ^2^(5) = 5.4, *p* > 0.05]. Based on such metric invariance of factor loadings of tasks on paradigm factors, we also tested a model with paradigm factors freely correlating. This resulted in satisfactory model fit [Model 2b: χ^2^(45) = 65.7, CFI = 0.97, RMSEA = 0.07, SRMR = 0.07] and high to very high latent correlations between paradigm factors (Table 3).

**Table 3:** Latent correlations of paradigm factors (Model 2b).

	**Complex span**	**Memory updating**	**Sorting span**	***n*-Back**
Complex span		0.78	0.48	0.69
Memory updating	1.06		0.64	0.80
Sorting span	0.67	0.61		0.69
*n*-Back	0.69	0.73	0.51	

Younger adults below the diagonal (N = 101), older adults above the diagonal (N = 103).

Constraining loadings of the paradigm factors on the working memory factor in the hierarchical model to be equal across age groups did not lead to significant loss of fit [Model 3: χ^2^(52) = 72.2, CFI = 0.97, RMSEA = 0.06, SRMR = 0.09, Δχ^2^(3) = 2.9, *p* > 0.05]. While in this model, unstandardized factor loadings on the working memory factor were constrained to be equal, the standardized loadings differed numerically. We therefore further tested whether the standardized loadings differed across age groups, including a set on non-linear constraints into Model 2. As the corresponding test was not significant [Δχ^2^(4) = 7.3, *p* > 0.05], we refrain from interpreting any apparent age group differences in the pattern of standardized loadings of the paradigm factors on the working memory factor and conclude that the paradigms do not differ reliably between age groups as indicators of working memory. Differences of standardized factor loadings within age groups were significant for the younger [Δχ^2^(3) = 15.0] but not for the older adults [Δχ^2^(3) = 5.5]. This indicates that, in younger adults, the working memory factor was more strongly defined by complex span and memory updating than by *n*-back and sorting span, while, in older adults, working memory was measured equally well with all paradigms.

In a final set of models, we included a factor of reasoning as a criterion that was predicted by the latent factor of working memory. Fit of this model was good [Model 4: χ^2^(108) = 139.4, CFI = 0.97, RMSEA = 0.05, SRMR = 0.10; see Figure 1]. The standardized regression path of reasoning on working memory was very high for younger (β = 0.84, SE = 0.06) as well as for older adults (β = 0.91, SE = 0.06). This model was compared to models in which reasoning was predicted with latent factors of the different paradigms singly. As shown in Table 4, none of the paradigms alone could explain as much variance in reasoning as the higher-order factor combining all paradigms. The highest amount of variance explained was found when using the sorting span factor as a predictor. Accordingly, a model with four correlated paradigm factors predicting reasoning resulted in sorting span being the strongest (and the only significant) unique predictor of reasoning. This might be due to the comparatively high complexity of this paradigm, in terms of both, storage and updating demands. First, the maximum number of elements to be held simultaneously in memory was 10 for alpha span and six for animal span, while it was only three for 3-back and four for memory updating. Second, at each updating step, only one element needs to be updated in the memory updating paradigm, three elements need to be updated in 3-back (but in a determined way, i.e., each elements needs to be shifted by one lag position), while the bindings of elements to rank positions need to be updated for several elements in a more complex way in the sorting span tasks (i.e., one has to first determine the correct rank position, and then update this one and shift all elements from this or larger rank positions). Given the central role of manipulating complex mental representations for reasoning ability (Oberauer et al., 2007), these aspects might explain the comparatively strong role of the sorting span tasks in predicting the reasoning factor in our analyses and calls for future work on the WM processes involved in this interesting paradigm.

**Table 4:** Prediction of reasoning with different latent factors.

	**Complex span alone**	**Memory updating alone**	***n*-Back alone**	**Sorting span alone**	**Correlated factors**	**Higher-order factor**
Latent R-square (younger adults/older adults)	0.51/0.16	0.59/0.50	0.21/0.65	0.64/0.74	0.79/0.82	0.71/0.83
χ^2^ (df)	19.1 (22)	10.1 (13)	14.8 (13)	8.7 (13)	100.4 (95)	139.4 (108)
RMSEA	0.00	0.00	0.04	0.04	0.02	0.05
CFI	1.00	1.00	1.00	1.00	1.00	0.97
SRMR	0.07	0.05	0.06	0.03	0.06	0.10

## DISCUSSION

Once measurement error and content-specific sources of variance were accounted for, latent factors of the complex span and the *n*-back paradigm correlated substantially, with *r* = 0.69 in both samples of younger and older adults. The high latent correlations of *n*-back, memory updating, and complex span tasks of working memory are in agreement with similar analyses by Wilhelm et al. (2013), who report even higher correlations between latent factors of the three paradigms, each represented by three tasks varying in task content. Their and our findings need to be contrasted to the meta-analysis of Redick and Lindsey (2013), who reported a correlation of *r* = 0.20. The difference in magnitude between the correlation found in this study and the meta-analytic correlation reported by Redick and Lindsey (2013) is most easily understood if we assume that the correlations summarized in the meta-analysis were systematically lowered by a combination of paradigm-specific variance, content-specific variance, and measurement error. In fact, the hierarchical model used here sheds light on the relative contributions of each of these sources of attenuation. For example, both reading span and numerical 3-back are valid indicators of their paradigm factors, and the two paradigms (complex span and *n*-back) are valid representations of the general working memory factor. Nevertheless, the shared variance due to working memory between these two tasks results from a multiplication of the corresponding four factor loadings (tasks on paradigm factors and paradigm factors on construct factor), which is 0.30 (= 0.48 × 0.99 × 0.74 × 0.85) for the younger and 0.26 (= 0.53 × 0.72 × 0.86 × 0.79) for the older adults. This explains why correlations in the range reported by Redick and Lindsey (2013) are not surprising for any combination of tasks that differ in paradigm, content, or both.

Our latent factors of complex span and *n*-back loaded highly on a general factor of working memory, which also comprised factors of the memory updating and the sorting span paradigms. Comparing these loadings across paradigms and across age groups indicated that all these paradigms are good operational definitions of working memory, but maybe not to same degree. Complex span and memory updating were close-to-perfect indicators of the general working memory factor for younger adults. *n*-Back and sorting span tasks had considerably lower loadings on the working memory factor. For older adults, the pattern was more homogenous with no significant differences between standardized factor loadings. As these findings are based on samples that are not excessively large and on particular operational definitions of tasks drawn out of a multitude of different operational definitions that one could think of, conclusions regarding the pros and cons of particular paradigms can at best be tentative with the present results. Instead, we would like to propose several general conclusions about task selection for working memory assessment that follow from the hierarchical psychometric perspective advocated in this article.

First, when one is interested in how individual differences in working memory are related to other constructs, like reasoning, it is advisable to represent working memory broadly with a heterogeneous selection of tasks drawn from different paradigms and using different content material, and to conduct analyses at the latent factor level with structural equation models (cf. Oberauer et al., 2008; Shelton et al., 2010; Wilhelm et al., 2013). As demonstrated by Little et al. (1999), capturing the centroid of a construct is more likely to be achieved by using indicators that differ on construct-irrelevant task attributes—even if this implies that they do not correlate highly with each other—than with indicators that are very similar, and therefore correlate highly, but cover only a relatively small sub-space of the space that fully defines the construct.

We checked whether this is the case in our data by running a permutation analysis, in which all 84 possible combinations of three working memory tasks selected from our battery of nine tasks were used to build a latent working memory factor with a given set of three selected tasks as indicators, which was then correlated with the latent factor of reasoning. We found that there was a negative correlation (*r* = –0.38 for younger and *r* = –0.35 for older adults; if restricted to models with good model fit as indicated by a RMSEA < 0.08: *r* = –0.43 for younger and *r* = –0.49 for older adults) between the estimate of the latent correlation of working memory and reasoning (range 0.46–1.02 for younger and 0.38–1.03 for older adults) and the average correlation among the three tasks (range 0.22–0.55 for younger and 0.26–0.56 for older adults). Given that the reliability of all tasks was relatively high, this means that the construct of working memory, when validated with its correlation to reasoning, was represented the better the more heterogeneous the selection of tasks was. In other words, selecting three tasks that are heterogeneous in terms of paradigm and content, and therefore only have relatively small correlations with each other, makes for a latent factor that correlates more highly with reasoning, and therefore better represents working memory, than latent factors based on a more homogenous selections of tasks.

Second, if one is interested in assessing working memory performance in specific individuals, latent factor approaches are less useful, but the same general arguments apply. Because individual differences in performance on any single working memory task are dominated by paradigm- and content-specific sources of variance, it is preferable to measure performance with a heterogeneous battery of tasks and use average performance (or some factor score estimate) as an indicator of working memory capacity. Depending on the population the individuals belong to (e.g., children, younger adults, older adults), different (combinations of) tasks might be preferable.

Third, if one is interested in investigating the mechanisms of working memory by applying experimental manipulations, formal mathematical models, and neuroscience methods, one typically has to choose a particular paradigm. This choice may be determined by theoretical as well as pragmatic reasons. Certain tasks might be picked because they are particularly well suited to investigate mechanisms such as switching the focus of attention, inhibiting no-more-relevant information, or interference due to cross-talk between elements in working memory. Other tasks might be given preference because they allow trial-based analyses in fMRI investigations or are easily explained to children. What we would like to caution against, however, is to equate a certain paradigm with the construct it is supposed to measure. Developing increasingly refined models to explain the processes of a particular paradigm carries the danger of ending up modeling task-specific aspects that are of limited relevance for understanding the theoretical construct of interest (cf. Salthouse, 1985). Cognitive psychology would profit a lot if researchers were attempting to test their theories not only on their preferred paradigms but in the entire domain of tasks that define a construct.

### Conflict of Interest Statement

The authors declare that the research was conducted in the absence of any commercial or financial relationships that could be construed as a potential conflict of interest.

## Footnotes

1 Please note that this collection of paradigms is by no means thought to be exhaustive. There are more working memory paradigms in the literature (like backward span) and new ones could be invented.
